# Restoration of vision in Kniest dysplasia patient characterized by retinal detachment with dialysis of the ora serrata: A case report

**DOI:** 10.1097/MD.0000000000036090

**Published:** 2023-11-24

**Authors:** Xinlei Zhu, Xiaoli Xing, Dongfang Li, Bin Yu

**Affiliations:** a Qingdao Eye Hospital of Shandong First Medical University, Qingdao, China; b State Key Laboratory Cultivation Base, Shandong Provincial Key Laboratory of Ophthalmology, Shandong Eye Institute, Shandong First Medical University & Shandong Academy of Medical Sciences, Qingdao, China; c Medical College, Qingdao University, Qingdao, China.

**Keywords:** case report, *COL2A1* gene, gene mutation, hereditary diseases, Kniest dysplasia

## Abstract

**Introduction:**

Congenital eye diseases have a significant impact on children and young adults. Retinal detachment associated with Kniest dysplasia represents the most severe ocular complication, which is challenging to diagnose and treat effectively. Genetic testing has emerged as an invaluable tool for diagnosing hereditary diseases.

**Case presentation:**

A 23-year-old male presented to our Ophthalmology Clinic with retinal detachment involving dialysis of the ora serrata in his left eye. High-throughput exon sequencing enabled a definitive diagnosis of Kniest dysplasia resulting from a mutation in the *COL2A1* gene. The patient subsequently underwent pars plana vitrectomy with silicone oil injection to reattach the retina. This surgical intervention successfully reattached the retina and restored vision to 20/25 in the affected eye.

**Conclusion:**

Retinal detachment represents the most serious ocular complication associated with Kniest dysplasia. To prevent permanent blindness, early diagnosis through genetic testing and regular ophthalmological examinations are imperative. Advances in genetic screening have improved the management of retinal detachment risk in Kniest dysplasia patients.

## 1. Introduction

Retinal detachment is a common ophthalmologic condition that severely threatens patient’s vision and quality of life. In contrast to adults, retinal detachment is rare in pediatric populations.^[1]^ However, retinal detachment etiologies in children are diverse, including hereditary vitreoretinopathies, retinopathy of prematurity, trauma, myopia, Coats disease, and others.^[1–4]^ Notably, congenital ocular diseases may manifest in young adulthood.^[5]^ Retinal detachments resulting from hereditary diseases can be insidious and may cause blindness without timely intervention.^[6]^ We present a clinically confirmed case of retinal detachment with dialysis of the ora serrata. The diagnosis is supported by definitive genetic evidence of a pathogenic mutation.

## 2. Case presentation

A 23-year-old male presented to our Ophthalmology Clinic with a 1-month history of blurred vision and 5-day history of deteriorating vision in his left eye. He was admitted for retinal detachment of the left eye. His medical history was significant for Pierre Robin syndrome, diagnosed in childhood based on physical examination at a local hospital. His parents had no similar medical history. Five years prior, he was evaluated for white pupil in his right eye and diagnosed with long-standing retinal detachment and cataract. At that time, the local clinicians believed the retinal detachment has lost the therapeutic value so he only underwent cataract phacoemulsification with intraocular lens implantation to improve cosmesis. Consequently, the opportunity for retinal reattachment surgery was missed and his right eye remained blind.

**Figure 1.**
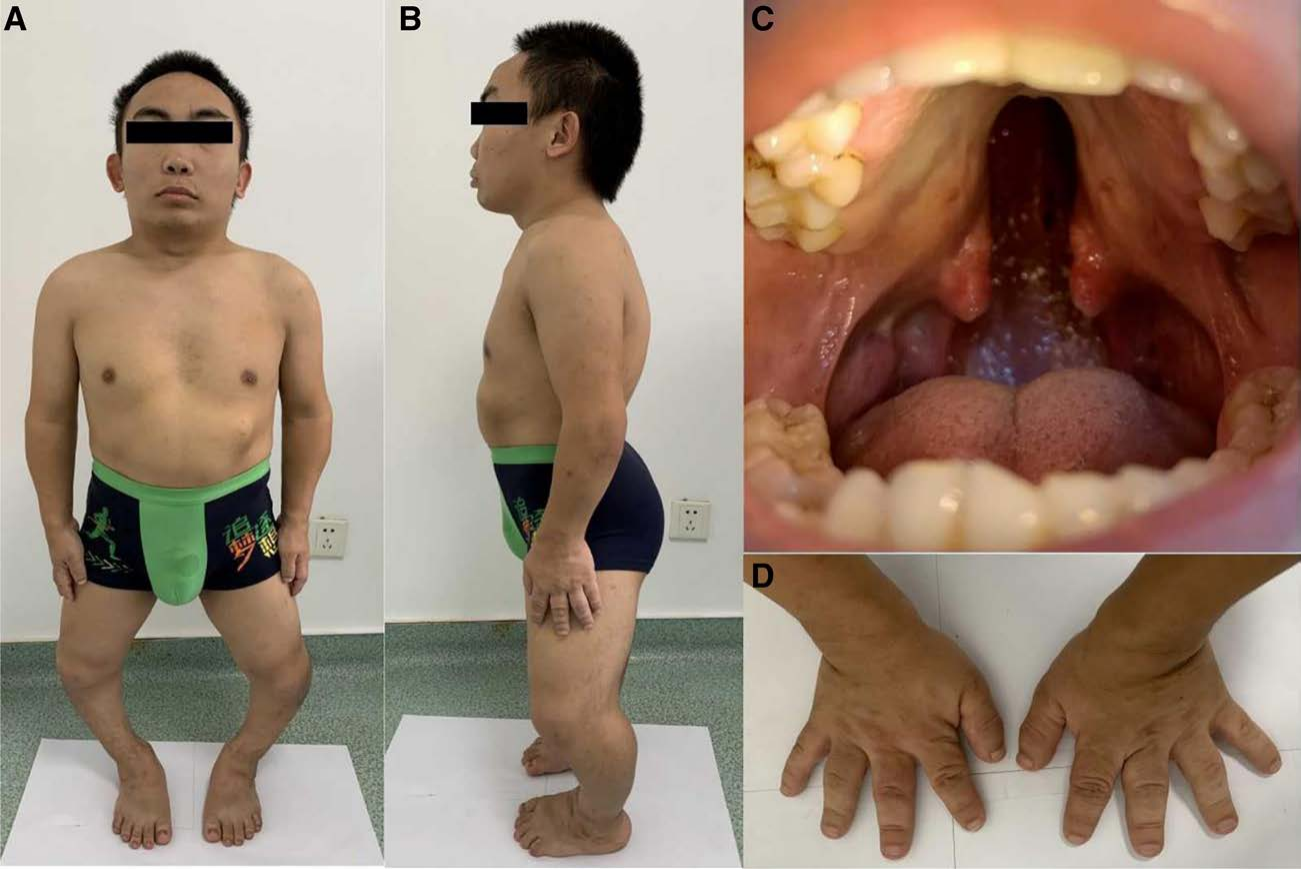
Physical examination revealed features consistent with Kniest dysplasia, including short stature, short limbs, prominent joints (A), flat nasal bridge, kyphoscoliosis, abdominal protrusion (B), cleft palate (C), and stubby, spoke-like fingers (D).

**Figure 2.**
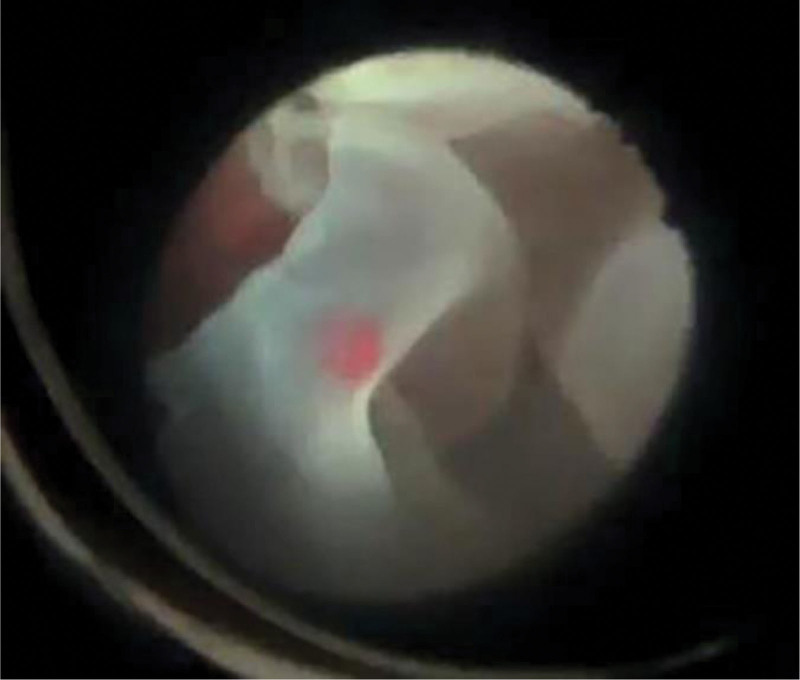
Fundus examination of the left eye revealed retinal detachment with near-circumferential dialysis of the ora serrata.

**Figure 3.**
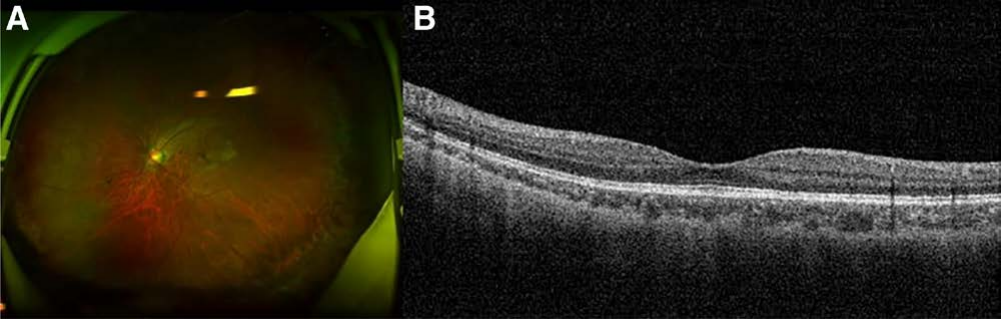
On last follow-up, the retina appeared reattached and reddish in color (A), and the macular foveal contour tended toward a more regular structure (B).

Despite normal intelligence, physical examination revealed short stature (71 cm), kyphoscoliosis, abdominal protrusion, shortened extremities, prominent joints, stubby and spoke-like fingers, enlarged forehead, flat nasal bridge, and cleft palate (Fig. 1). He also had high myopia and hearing impairment. Ophthalmologic examination showed no light perception in the right eye, with proliferative membranes in the vitreous cavity and closed-funnel retinal detachment. In the left eye, uncorrected visual acuity was hand motion with no improvement on correction. Intraocular pressure was 6 mm Hg. Slit lamp examination of the left eye demonstrated corneal endothelial pigment deposits from 3 to 9’o clock, inhomogeneous cataract with super-temporal partial defect, and tremor. Due to cataract, the left fundus could not be clearly visualized. Ultrasound biomicroscopy (UBM) showed anterior chamber angle narrowing, lens subluxation, and epichoroidal spaces leakage in the left eye. B-scan ultrasonography revealed retinal and choroidal detachment in the left eye, further confirming the diagnosis. On admission, we performed pars plana vitrectomy combined with lensectomy. Intraoperatively, we observed complete 360° dialysis of the ora serrata with infolding of the retina into a flower bud appearance (Fig. 2). The peripheral retina was shrunken and undulated, requiring radial and segmental retinotomies to relieve traction. With perfluorocarbon liquid assistance, the retina was successfully reattached and the ora serrata consolidated with laser. After perfluorocarbon-silicone oil exchange, the operation was completed. Best corrected visual acuity (BCVA) was 20/200 one day postoperatively, with attached retina under silicone oil tamponade. At 1 month, BCVA improved to 20/80 with restored retinal color and improving foveal contour. BCVA then stabilized at 20/25 by 2 months. Silicone oil was removed at 3 months. At the 5-month follow-up after silicone oil removal, BCVA remained stable at 20/25 with attached retina (Fig. 3A and B). BCVA was measured using the international standard visual acuity chart and converted to Snellen equivalent. Intraocular pressure was maintained within the normal range after each surgery.

**Figure 4.**
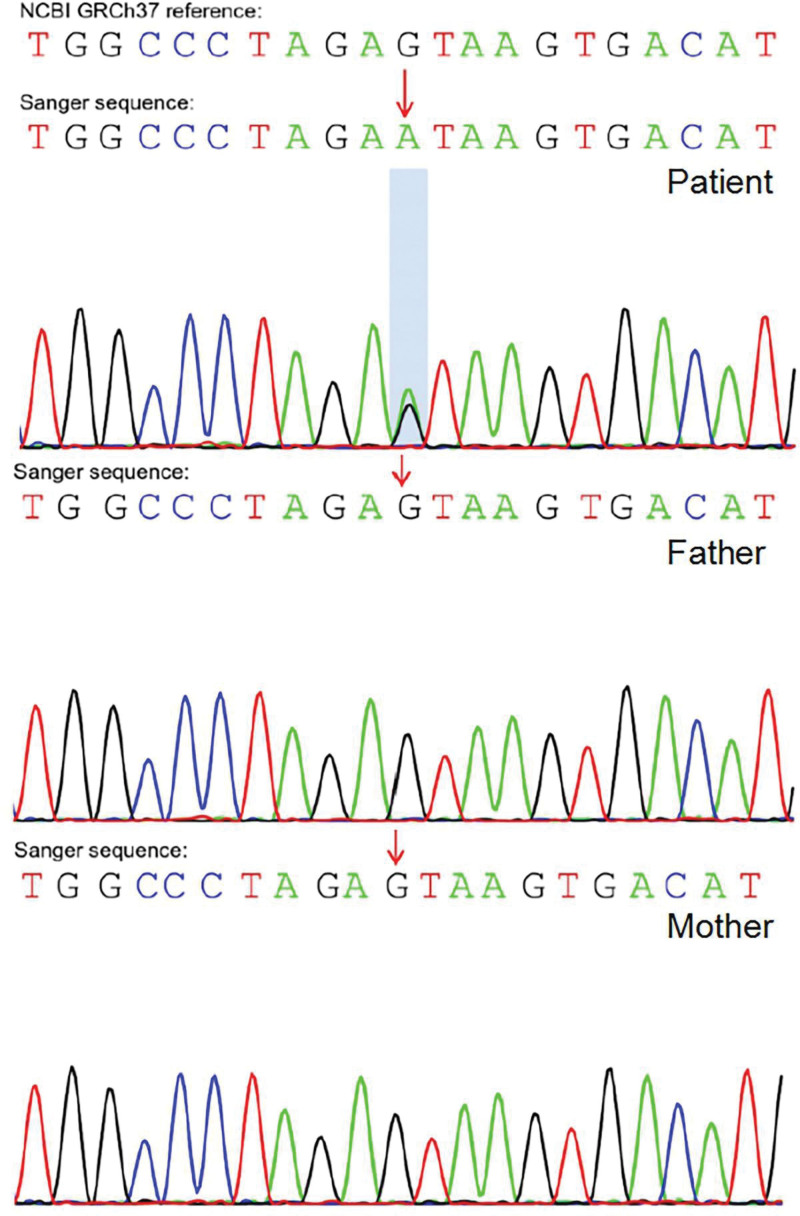
Genetic analysis revealed a heterozygous splicing mutation in the *COL2A1* gene (NM_001844), exon c3489 + 1G>A in the patient. This mutation was absent in the parents, confirming it arose de novo.

Based on the ocular findings and systemic manifestations, a definitive diagnosis could not be made initially. However, a rare hereditary syndrome was suspected. Therefore, we recommended genetic testing for the patient and his phenotypically normal parents after obtaining informed consent. We were granted permission to report personal images and medical history. This study was approved by the Ethics Committee of Qingdao Eye Hospital Affiliated with Shandong First Medical University and adhered to the Declaration of Helsinki. Whole genome DNA from the patient and parents underwent high-throughput exon sequencing of 4503 common pathogenic genes at the Beijing Giantmed Medical Diagnostics Lab, including single nucleotide variations and indels in the coding regions. Genetic analysis revealed a heterozygous splicing mutation in the *COL2A1* gene (NM_001844), specifically exon c3489 + 1G > A, in the patient that was absent in the parents (Fig. 4). This mutation has not been previously reported in Online Mendelian Inheritance in Man or the Human Gene Mutation Database.

## 3. Discussion and conclusion

The *COL2A1* gene is located on human chromosome 12q13.11-q13.2 and contains 54 exons. It encodes the precursor protein for type II collagen, known as type II procollagen.^[7]^ Type II collagen, also termed cartilage collagen, is the predominant collagen synthesized by chondrocytes and is also present in the vitreous. Therefore, defects in *COL2A1* lead to a phenotypically diverse group of disorders collectively known as type II collagenopathies or *COL2A1*-related disorders, with an estimated incidence of 1.6 per 10,000.^[8]^ These conditions typically demonstrate an autosomal dominant inheritance pattern and have multisystem manifestations, including skeletal, orofacial, auditory, and ocular features.^[8]^

Kniest dysplasia is a rare skeletal-chondrogenic dysplasia first described by Wilhelm Kniest in 1952. The disease arises from mutations in the *COL2A1* gene encoding type II collagen and demonstrates an autosomal dominant inheritance pattern. Characteristic features include skeletal and craniofacial abnormalities.^[9,10]^ Skeletal manifestations consist of disproportionate dwarfism, short trunk and small pelvis, kyphoscoliosis, short limbs, prominent joints, and premature osteoarthritis resulting in restricted mobility. Craniofacial features comprise midface hypoplasia, cleft palate, early-onset myopia, retinal detachment, and hearing loss.^[9,10]^

To date, over 400 likely pathogenic variants have been described in the *COL2A1* gene (human gene mutation database). We report a clinically confirmed case of Kniest dysplasia with a clear gene mutation. The patient presented to our Ophthalmology Clinic for retinal detachment in the left eye. He had been diagnosed with Pierre Robin syndrome during childhood. However, his systemic and ocular manifestations were concerning. Through high-throughput exon sequencing, the correct diagnosis of Kniest dysplasia was made. Additionally, the identified *COL2A1* gene mutation represents the first report of this particular variant in Kniest dysplasia.

Ocular manifestations of hereditary diseases are often overlooked. The patient’s right eye was completely blind due to missed surgical intervention. Fortunately, vision in the left eye was restored through elaborate surgery. If childhood eye examinations had been performed regularly after being informed of potential accompanying ocular disease, his condition may have been less severe on presentation to our Ophthalmology Clinic. Previous studies have recommended complete ophthalmologic exams for patients clinically diagnosed with Kniest dysplasia.^[11]^ Congenital non-progressive high myopia, vitreous abnormalities, and retinal detachment are important ocular features of this condition.^[9]^ Additionally, other type II collagen disorders may demonstrate similar ophthalmic findings, thus regular ophthalmologic follow-up is advised.^[12]^

Unlike other cases of Kniest dysplasia, this patient was diagnosed with Pierre Robin syndrome in early childhood. Pierre Robin syndrome was first described in 1923 by the French stomatologist Pierre Robin, who reported the association between micrognathia, glossoptosis, and cleft palate.^[13]^ Previous studies have demonstrated that Robin sequence can be associated with various genetic syndromes, most commonly Stickler syndrome and 22q11 deletion syndrome (22q11 DS).^[14]^ The presence of ocular, skeletal or auditory abnormalities in children with Pierre Robin Sequence (PRS) should prompt suspicion for an underlying genetic syndrome. Compared to isolated PRS, additional features of Kniest dysplasia include hearing loss, retinal detachment, and progressive ocular complications that may eventually lead to blindness, as exemplified in this patient’s right eye.^[15]^ Early identification of the genetic syndrome underlying PRS is crucial for prompt ophthalmologic referral to monitor for myopia, retinal detachment, and prevention of visual complications.^[16]^ Given the long-term risks of retinal detachment, hearing loss, and osteoarthritis with Kniest dysplasia, long-term multidisciplinary follow-up and management in ophthalmology, orthopedics, and otolaryngology are warranted in conjunction with treatment of PRS features.^[16]^

In conclusion, retinal detachment represents the most severe ocular complication in Kniest dysplasia. Treatment is challenging given the earlier age of onset, vitreous degeneration, presence of multiple large retinal tears, and delays in diagnosis. To prevent blindness, close follow-up with regular ophthalmologic exams for early diagnosis and intervention is essential. Genetic testing to identify *COL2A1* mutations can aid in establishing the diagnosis. If retinal detachment occurs, urgent pars plana vitrectomy to reattach the retina is an effective treatment approach.

## Author contributions

**Conceptualization:** Xiaoli Xing, Dongfang Li, Bin Yu.

**Data curation:** Xinlei Zhu, Dongfang Li.

**Formal analysis:** Xinlei Zhu.

**Investigation:** Xinlei Zhu, Xiaoli Xing.

**Project administration:** Xiaoli Xing.

**Visualization:** Dongfang Li.

**Writing – original draft:** Xinlei Zhu.

**Writing – review & editing:** Xinlei Zhu, Bin Yu.
